# Laparoscopic cholecystectomy – is there a need to convert?

**DOI:** 10.4103/0972-9941.16528

**Published:** 2005-06

**Authors:** Kuldip Singh, Ashish Ohri

**Affiliations:** Department of Surgery, Dayanand Medical College and Hospital, Ludhiana, Punjab, India

**Keywords:** Laparoscopic cholecystectomy, conversion

## Abstract

**Introduction:**

The difficult gallbladder is the most common ‘difficult’ laparoscopic surgery being performed by general surgeons all over the world and the potential one that places the patient at significant risk. We present our experience of 6147 cases since January 1993 in a single center with respect to conversion to open cholecystectomy.

**Methods:**

Patients who underwent laparoscopic cholecystectomy (LC) from January 1993 to December 2004 were analyzed. The cases were analyzed in relation to conversion rate to open surgery, factors affecting the conversion, and completion rate of LC. Patients having absolute contraindications to LC like cardiovascular and pulmonary disease were not included in the study.

**Results:**

Out of 6147 cases, 1518 patients (21.5%) were identified as difficult cases. Laparoscopic cholecystectomy was successfully completed in 6125 patients with a completion rate of 99.6%. Laparoscopic procedure had to be converted to the open procedure in 22 patients with a conversion rate of 0.36% of the total LCs performed and 1.66% of the difficult cases. Conversion had to be done due to several reasons.

**Conclusion:**

It can be reliably concluded that LC is the preferred method even in the difficult cases. Our study emphasizes that although the rate of conversion to open surgery and complication rate are low in experienced hands the surgeon should keep a low threshold for conversion to open surgery and it should be taken as a step in the interest of the patient rather than be looked upon as an insult to the surgeon.

## INTRODUCTION

Laparoscopic cholecystectomy (LC) has almost replaced open cholecystectomy as the therapeutic modality in the treatment of symptomatic gallstones. The difficult gallbladder is the most common ‘difficult’ laparoscopic surgery being performed by general surgeons all over the world and the potential one that places the patient at significant risk. A number of published clinical series[1]–[4] including this, emphasize the promising role laparoscopy is playing in treating gallbladder disease. In the beginning of LC, patients like acute cholecystitis, empyema, gangrenous gallbladder, cirrhotic patients, and Mirizzi syndrome were contraindicated because of high risk of complications and conversion rate.[2][3] After more than 10–12 years of learning and understanding the technique, and surgeons gaining more expertise, we thought it imperative to reassess the feasibility of these complicated cases laparoscopically in terms of conversion rate. We present our experience of 6147 cases since January 1993 in a single center with respect to conversion to open cholecystectomy.

## MATERIALS AND METHODS

Patients who underwent LC from January 1993 to December 2004 were analyzed. Ultrasonography (USG) was the mainstay for the preoperative diagnosis of gallstone disease [Crade's criteria with common bile duct (CBD) status].[5] Indications for LC included cholelithiasis and biliary colic (cholecystitis), symptomatic gallbladder polyps, gallstone pancreatitis, calcified gallbladder, large gallstone (> 2 cm), nonfunctioning gallbladder, and chronic typhoid carrier.[6] Our policy has been to take up all the cases which merit cholecystectomy and are fit to undergo laparoscopy. Patients clinically having pain, rigidity, guarding and palpable lump or Phlegmon were taken up for LC once these clinical parameters resolved. Common bile duct stones were suspected preoperatively based on raised alkaline phosphatase, dilated CBD on USG, stone visible in CBD on USG and history of jaundice. These patients were subjected to preoperative ERCP and after CBD clearance were included in the study and those who failed endoscopic clearance were subjected to open surgery and were not included in the study. We did not subject the patients to intraoperative cholangiography (IOC). The definition of difficult cholecystectomy is not consistent. We defined difficult cholecystectomy as (1) dense adhesions at the triangle of Calot (frozen triangle of Calot prohibiting proceeding laparoscopically without risk), (2) contracted and fibrotic gallbladder, (3) previous abdominal surgery, (4) gangrenous gallbladder, (5) acutely inflamed gallbladder, (6) empyema gallbladder (including Mirrizi syndrome Type II), and (7) cholecystogastric or cholecystoduodenal fistula. The cases were analyzed in relation to conversion rate to open surgery, factors affecting the conversion and completion rate of LC. Patients having absolute contraindications to LC like cardiovascular and pulmonary disease were not included in the study.

## RESULTS

Laparoscopic cholecystectomy was performed in 6147 patients during January 1993–December 2004, out of which 2124 were males and 4023 were females with an average age of 48.6 years (range 22–84 years). Out of 6147 cases, 1325 patients (21.5%) were identified as difficult cases. Laparoscopic cholecystectomy was successfully completed in 6125 patients with a completion rate of 99.6%. Laparoscopic procedure had to be converted to the open procedure in 22 patients with a conversion rate of 0.36% of the total LC performed and 1.66% of the difficult cases. Conversion had to be done due to several reasons [Table 1]. Out of 12 patients who had dense adhesions in the triangle of Calot and unclear anatomy, two patients (16.7%) required conversion to open surgery. A total of 280 patients had contracted and fibrotic gallbladder, of which four patients (1.4%) had to be converted. Out of 640 patients having acutely inflamed gallbladder, four patients (0.62%) had to be converted. Only two patients (5.71%) having dense adhesions due to previous upper abdominal surgery out of 35 patients had to be converted. Cholecystectomy was present in 12 patients of which one patient (8.3%) had to be converted. Gangrenous gallbladder was the reason for conversion in two patients (4.25%) of 47 patients. There were six (2.05%) conversions in a group of 293 patients having empyema of gallbladder. Six patients had cholecystogastric or cholecystoduodenal fistula and LC was successfully done in five patients and only one patient (16.66%) had to be converted to open. Only three conversions were enforced due to intraoperative CBD injury. Two patients had the right hepatic duct clipped due to misidentification of the right hepatic duct as the cystic duct (Strassberg Type B). Two patients having contracted gallbladders and one patient with dense adhesions in the Calot's triangle and common hepatic duct area suffered a lateral injury during dissection (Starssberg Type D). All the injuries were identified intraoperatively and were managed in the same sitting. It has been our policy to keep a drain after LC for 24 h. Eight patients had increased postoperative bile leak, which spontaneously stopped within a period of 1 week, however, the source of the bile leak could not be identified. We categorize them as minor leak either from the gallbladder bed or from accessory cystic duct (Strassberg Type A). There was no mortality. Conversions were more in the first 3000 cases as compared to the next 3000 [Table 2]. Operating time varied from 30 min to 2.5 h (mean 45 min). Age, sex, previous abdominal surgery, and most importantly surgeon's experience were found as important determinants of successful outcome.

**Table 1:** Indication for conversion to open cholecystectomy (*n* = 22)

Indication for conversion	Total number of cases (*n* = 1325)	Conversions (*n* = 22)	Percentage
Dense adhesions at Calot's triangle	12	2	16.7%
Contracted gallbladder	280	4	1.4%
Acutely inflamed gallbladder	640	4	0.62%
Gangrenous gallbladder	47	2	4.25%
Previous upper abdominal surgery	35	2	5.71%
Previous cholecystectomy	12	1	8.3%
Empyema gallbladder	293	6	2.05%
Cholecystogastric and cholecystoduodenal fistula formation	6	1	16.6%

**Table 2:** Difficult cases and conversions

Total cases Percent(%)	Difficult cases	Conversions
0–1000	320	8 2.5
1000–2000	240	6 2.5
2000–3000	310	5 1.6
3000–4000	185	0 0
4000–5000	160	1 0.6
6000–6147	110	2 1.8

## DISCUSSION

Philip Mouret in 1987 was the first to remove the gallbladder successfully through an unmagnified mechanical rigid pipe without doing laparotomy. Dubois is credited for popularizing LC.[7] Initially, the complication rate with LC was high but as the experience has grown, it has reached a remarkably low level at 2.0–6.0%.[6] Although the incidence is still higher than the incidence after open surgery. Since 1990 many surgeons have attempted LC with reasonable success in difficult cases.[2]–[4][8] Their results indicated that extensive experience with both open and laparoscopic biliary tract surgery is the most important ingredient of a successful outcome in the setting of difficult cases.

The clinical profile of a patient can predict a difficult gallbladder surgery. Based on our experience we feel that even in a patient anticipated to have a difficult gallbladder one can complete the procedure laparoscopically. Hence our policy has been to take up all the cases fit to undergo laparoscopy for LC. Conversion to open surgery is not visualized as a complication, rather a matter of sound surgical judgment as patient safety is of foremost importance. We had to modify our technique by (a) placing additional trocars to facilitate liver and duodenal retraction, (b) by aspirating the gallbladder through the 5 mm subcostal trocar, or (c) by using a 10 mm toothed grasper for grasping thick walled gallbladders. In cases with acute inflammation of the gallbladder, a peanut swab held in a grasper used for blunt dissection allowed planes to be opened up with greater ease and safety. Aspiration of the distended gallbladder allows confirming the diagnosis and at the same time makes the handling of the gallbladder easier during the subsequent dissection.

In our experience the overall conversion rate was 0.36% of the total LCs performed and 1.66% of the difficult cases, which is in accordance with the literature (2–6%).[9] Dense adhesions at the triangle of Calot's was the most common reason for conversion to open surgery (16.7%)[9] with gastric and duodenal fistula (16.6%) being the second most common reason for conversion out of the difficult cases. Only three conversions were enforced due to CBD injury all of which were identified intraoperatively and managed in the same sitting. All the patients having increased bile leak postoperatively were managed conservatively and the leak ceased spontaneously within 7 days. We attribute our low rate of conversion to the fact that we have been operating with the same team and adhering to the basic principles of laparoscopic surgery. Since the rate of conversion in patients with acutely inflamed gallbladder was 0.62%, we recommend LC in acute cholecystitis where feasible as has been reported in the literature.[10] We still believe from our experience that within 72 h of symptoms the tissue planes are edematous and inflamed but are easier to dissect, having no adhesions at all. But after 72 h, the tissue becomes more friable and becomes dangerous and risky to dissect till 3–4 weeks time when inflammation subsides and fibrosis sets in. Although we took up the patients for surgery even after 72 h in 22 cases and could complete them without conversion the intraoperative level of difficulty was high and it was risky and the mean operative time was much longer (85 *Vs* 45 min).

## CONCLUSION

It can be concluded that LC is the preferred method even in difficult cases. Our study emphasizes that although the rate of conversion to open surgery and the complication rate are low in experienced hands the surgeon should keep a low threshold for conversion to open surgery and it should be taken as a step in the interest of the patient rather than be looked upon as an insult to the surgeon.
